# SegR3D: A Multi-Target 3D Visualization System for Realistic Volume Rendering of Meningiomas

**DOI:** 10.3390/jimaging11070216

**Published:** 2025-06-30

**Authors:** Jiatian Zhang, Chunxiao Xu, Xinran Xu, Yajing Zhao, Lingxiao Zhao

**Affiliations:** 1 School of Biomedical Engineering (Suzhou), Division of Life Sciences and Medicine, University of Science and Technology of China, Hefei 230026, China; zjt20174025@mail.ustc.edu.cn (J.Z.); feimos@mail.ustc.edu.cn (C.X.); xuxinran@mail.ustc.edu.cn (X.X.); 2 Suzhou Institute of Biomedical Engineering and Technology, Chinese Academy of Sciences, Suzhou 215163, China; 3 Department of Radiology, Huashan Hospital, Fudan University, 12 Wulumuqi Rd. Middle, Shanghai 200040, China; zhaoyj92918@163.com

**Keywords:** medical visualization, semi-supervised learning, image segmentation, realistic volume rendering

## Abstract

Meningiomas are the most common primary intracranial tumors in adults. For most cases, surgical resection is effective in mitigating recurrence risk. Accurate visualization of meningiomas helps radiologists assess the distribution and volume of the tumor within the brain while assisting neurosurgeons in preoperative planning. This paper introduces an innovative realistic 3D medical visualization system, namely SegR3D. It incorporates a 3D medical image segmentation pipeline, which preprocesses the data via semi-supervised learning-based multi-target segmentation to generate masks of the lesion areas. Subsequently, both the original medical images and segmentation masks are utilized as non-scalar volume data inputs into the realistic rendering pipeline. We propose a novel importance transfer function, assigning varying degrees of importance to different mask values to emphasize the areas of interest. Our rendering pipeline integrates physically based rendering with advanced illumination techniques to enhance the depiction of the structural characteristics and shapes of lesion areas. We conducted a user study involving medical practitioners to evaluate the effectiveness of SegR3D. Our experimental results indicate that SegR3D demonstrates superior efficacy in the visual analysis of meningiomas compared to conventional visualization methods.

## 1. Introduction

Medical visualization is crucial for assisting physicians in analyzing diseases [1,2], particularly for different lesions. Meningiomas are the most common primary intracranial tumors in adults. Most meningiomas can be surgically resected to reduce the risk of recurrence [3]. Visualization of meningiomas helps radiologists assess the location and volume of the tumor within the brain and facilitates neurosurgeons’ surgery planning.

However, traditional medical 3D visualization methods struggle to effectively distinguish lesion areas from normal tissue due to overlapping pixel value ranges between tumor and non-tumor regions [1,4]. Conventional methods usually fail to accurately identify lesion areas and highlight them automatically. For a more effective analysis of tumors’ 3D structures, segmentation of tumors is frequently required. In recent years, with the development of deep learning, automatic segmentation technique has been significantly improved [5]. However, the technique relies on meticulously annotated data of high quality, which is expensive and time-consuming to acquire. More and more deep learning methods have adopted semi-supervised learning (SSL) strategies to reduce the dependence on annotations [6,7,8,9]. Therefore, we embed the SSL-based segmentation method into our visualization system to reduce the cost of manual annotations.

The visualization of segmentation results is also crucial. Traditional approaches involve extracting meshes from segmented masks, yet they struggle to analyze tumor positions across the brain. Direct volume rendering (DVR) can reveal the internal structure of volume data and stands as a pivotal technique in medical visualization [1,10,11,12]. It aids in displaying richer hierarchical details in the rendered results. Existing DVR technologies [10,13] primarily focus on various color effects, with little attention given to how to highlight regions of interest for the user. Most of these technologies focus solely on assigning material properties based on non-semantic features of volumetric data, without considering semantic information. For volumetric data that includes pre-extracted structures of interest, there is a lack of research on how to better display the shape of these structures and their relative positional relationships with surrounding tissue structures. Particularly for the target users (medical professionals), an important research focus is how to achieve optimal visualization results with minimal operation, without the need for a background in visualization theory.

**Figure 1:**
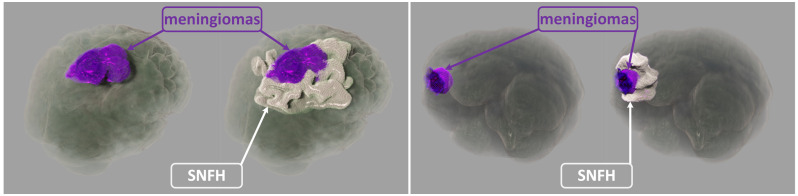
The visualization of meningiomas (encompassing both enhancing tumor and non-enhancing tumor core regions) and the surrounding non-enhancing T2/FLAIR hyperintensity (SNFH) from BraTS 2023 dataset [21] using the SegR3D system.

In this paper, our realistic rendering system SegR3D is introduced. It aims to enhance tumor visualization to assist the physician in surgical planning. Two examples of the visualization results using SegR3D are provided in Figure 1. Our main contributions are as follows: (1) We present an interactive visualization system that integrates a segmentation pipeline to obtain lesion regions of meningiomas. Our system offers a visualization method that fuses the original medical images and the segmentation results. (2) An SSL-based segmentation model is proposed to acquire the lesion area of meningiomas, named uncertainty correction pyramid model based on probability-aware cropping (UCPPA). This model offers a simple training process, as it eliminates the need for multiple forward passes [14], thereby enhancing SegR3D’s inference efficiency [9]. The probability-aware weighted random cropping employs a finite set of labels to construct a cropping probability mask. The mask is used to extract more sub-volumes from the lesion regions in both labeled and unlabeled images, optimizing the use of data. (3) We propose a novel importance transfer function to enhance the rendering outcome by emphasizing areas that we consider to be more significant for identifying tumors. We integrate advanced illumination techniques to enhance the stereoscopic quality of the rendering outcome [10,11,15,16,17,18]. The spatial partitioning acceleration technique is used to enable real-time interaction for users [19,20].

In our SegR3D system, users can simply drag and drop data onto the GUI, and the segmentation algorithm automatically executes, providing visual results within 5 s. Users can choose between real-time denoising [22] or progressive convergence to obtain noise-free visualization results. The interactive frame rate can reach over 80 frames per second on an Nvidia 2080Ti. Users only need to adjust the importance values for different regions, without requiring any further knowledge of DVR or transfer functions, to obtain visualizations that highlight specific areas. The interaction is intuitive and user-friendly.

Based on the evaluation conducted by multiple clinicians, our system shows outstanding performance in tumor analysis and surgical planning compared to conventional methods. It can be used as a useful tool for medical visualization.

## 2. Materials and Methods

We used a publicly available dataset from the Brain Tumor Segmentation 2023 Meningioma Challenge (BraTS2023-MEN) [21]. This dataset consists of 1650 cases from six medical centers, with an annotated training set of 1000 cases, each providing multiparametric MRI (mpMRI) (T1-weighted, T2-weighted, T2-FLAIR, T1Gd) and ground truth annotations by radiologists with 10+ years of experience. The annotations consist of non-enhancing tumor core (NETC), enhancing tumor (ET) and SNFH.

In our experiments, NETC and ET were categorized as meningiomas, representing the primary surgical targets for gross total resection. We divided the 1000 T2-FLAIR series into three subsets: 666 for training, 134 for validation, and 200 for testing. Patient demographics are given in Table 1.

### 2.1. The Realistic 3D Medical Visualization System

**Figure 2:**
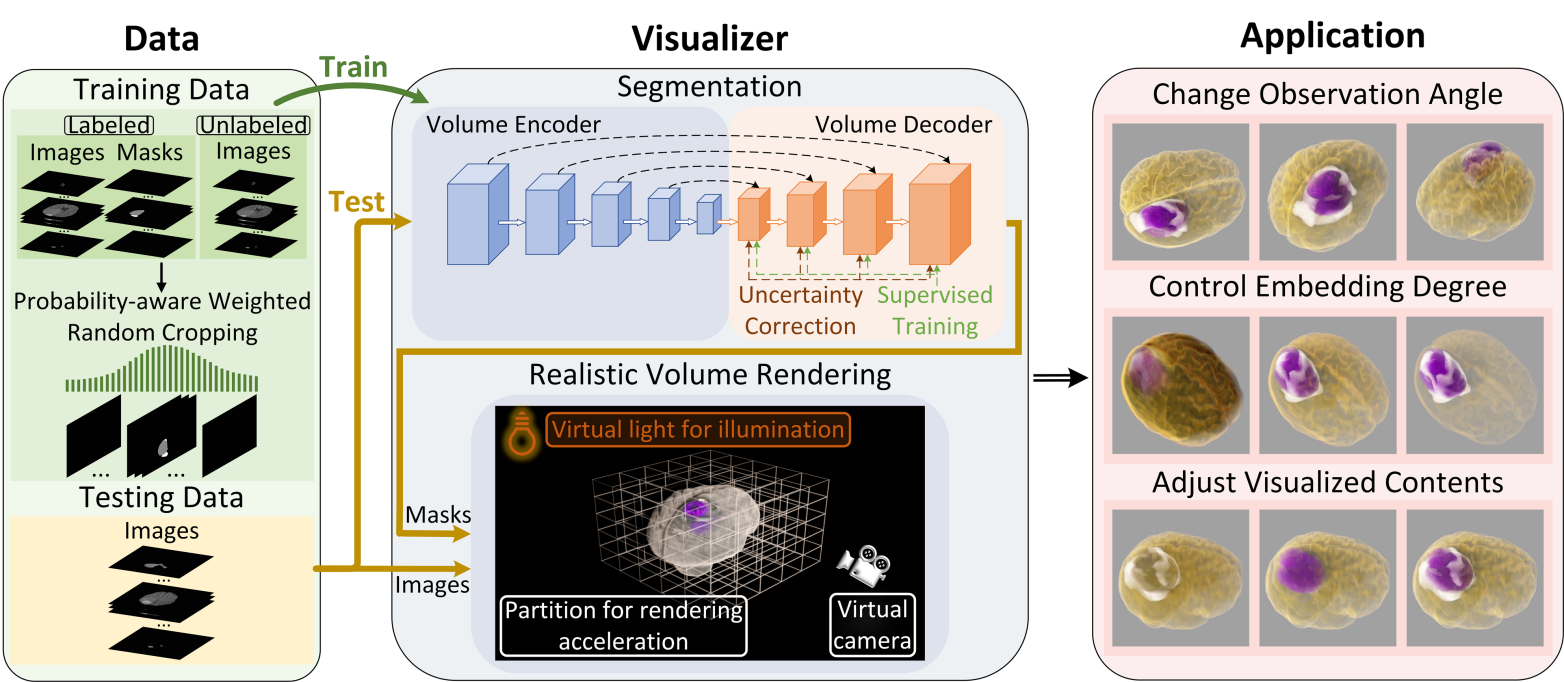
The framework of SegR3D. Medical images are input into the segmentation model (UCPPA) to acquire segmentation results. Subsequently, both the medical images and masks are provided as input to the volume renderer. SegR3D offers interactive volume visualization effects. Users can adjust the target object’s appearance and observation direction.

In this section, we describe the realistic 3D medical visualization system: SegR3D. It utilized a semi-supervised segmentation model and interactive realistic rendering for the visualization analysis of lesion regions, as illustrated in Figure 2.

#### 2.1.1. Semi-Supervised Segmentation Model

**Uncertainty Correction Pyramid Model.** The SegR3D system adopted a 3D semi-supervised segmentation method for outlining lesion areas of meningiomas in MRI. The training set was divided into a labeled data set $D_{l}={\left\{\left(x_{i},y_{i}\right)\right\}}_{i=1}^{N_{l}}$ and an unlabeled data set $D_{u}={\left\{x_{i}\right\}}_{i=1}^{N_{u}}$, where $x_{i}\in R^{H\times W\times D}$ was the input volume and $y_{i}\in {\left\{0,1,2\right\}}^{H\times W\times D}$ was the ground-truth annotation (2 foreground categories). We referred to the design of uncertainty rectified pyramid consistency (URPC) [9]. The auxiliary segmentation headers were added to V-Net [23] decoders at different resolution levels. For the input image $x_{i}$, the network generates a set of segmentation results of different scales. The results were resized to match the dimensions of $x_{i}$ by upsampling, yielding the sequence $\left[p_{1},p_{2},\ldots ,p_{s}\right]$. For the inputs with labeled *y*, the supervised optimization objective was the combination of two loss functions and can be formulated as:

(1) $$L_{s}=\frac{1}{S}\sum_{s=1}^{S}\frac{1}{2}\left(L_{ce}\left(p_{s},y\right)+L_{Dice}\left(p_{s},y\right)\right)$$

where $L_{ce}$ is the robust cross-entropy loss, and $L_{Dice}$ is the soft dice loss.

For unlabeled data, we calculated the loss $L_{u}$ through a scale-level uncertainty-aware approach [9]. The total optimization objective includes the supervised loss and the unsupervised loss, which is formulated as:

(2) $$L_{total}=L_{s}+\lambda L_{u}$$

where λ is a widely-used time-dependent Gaussian warming up function [9]. It can be used to control the trade-off between supervised and unsupervised losses, which is defined as:

(3) $$\lambda \left(t\right)=w_{max}e^{\left(-5{\left(1-\frac{t}{t_{max}}\right)}^{2}\right)}$$

where $w_{max}$ means the final regularization weight, *t* denotes the current training step, and $t_{max}$ is the maximal training step.

**Probability-Aware Weighted Random Cropping.** Most meningiomas are small in comparison to the brain and appear in a limited number of sequences in MRI. Inspired by the work of Lin et al. [24], we develop the probability-aware weighted random cropping strategy to make the model focus more on the lesion region. For each labeled image $x_{i}\in R^{H\times W\times D}$, we establish a list $l_{i}$ with the length of *D*, where each element’s index represents the starting point on the depth axis for cropping. An element at index *j* in list $l_{i}$ is assigned a value of 1 only if the cropping window starting at *j* contains more than *k* voxels labeled as foreground. Then these lists are aggregated across all labeled images to form a list $L=\sum_{i=1}^{N_{l}}l_{i}$. The element values in *L* represent the probabilities of the elements, and the indexes of the elements in *L* are selected for cropping through a weighted random selection process. Empirically, we set the threshold *k* to 50 in our experiments.

#### 2.1.2. Visualization

Our research work focused on three key aspects for the visualization of meningiomas in MRI: (1) We integrated and displayed lesion segmentation results with the original medical images. This allows physicians to easily identify lesion areas and perform an objective volumetric assessment of meningiomas. (2) We incorporated advanced illumination and shadowing to enhance the 3D sense. (3) We emphasized efficient real-time computation to meet interactive requirements.

**Visualization of Non-Scalar Data.** Each pixel of the input data for the renderer comprised a 2D vector v, representing the original medical image pixel value and the mask value generated by the segmentation pipeline. A 2D transformation function T:v→m was defined, which mapped voxel values to material attributes [1]. These material attributes commonly used in realistic DVR include opacity, phase function coefficients, albedo, and smoothness [25].

We designated the foreground in the segmentation results as “important regions”. In visualization, it is imperative to prevent unimportant regions from impacting the observation of important regions in the rendered results. To address this issue, we proposed a novel importance transfer function as the second dimension of *T*. The transfer function can be used to translate the mask value g∈[0,1] into the importance value *I*, which is defined as:

(4) $$I=\frac{\left(1-e^{-ag}\right)\left(1+e^{-a}\right)}{\left(1+e^{-ag}\right)\left(1-e^{-a}\right)}$$

The material attribute values, namely opacity, smoothness and albedo, were readjusted based on importance value. The new attribute values were obtained by multiplying them with *I*. If the importance value *I* was lower than 1, each of the attribute values was reduced by *I*. *I* represented a nonlinear transformation of *g*, providing finer control over regions with higher importance. In this equation, a(a>1) controls the level of precision, with higher values enabling more detailed control over regions of greater importance. The importance transfer function was applied to smoothness, albedo and opacity to highlight regions of interest. The effects of this function were discussed in Section 3.2.

**Realistic DVR.** Hybrid volumetric scattering and surface scattering model [25,26] was employed as the shading model. Utilizing this shading model can yield rendering outcomes with realistic material appearances. The radiative transfer equation (RTE) simulates the process of light propagation within a volumetric space, constituting a technique for achieving realistic DVR [17,25,27]. The Monte Carlo-based null-scattering algorithm [27] represents an advanced method for solving the RTE and was used in SegR3D.

**Acceleration Structure.** The maximum density value in the volumetric space is higher than the density in the majority of regions. This results in a significantly reduced average sampling step length [19,20,27], thus greatly impacting sampling efficiency. In SegR3D, the volumetric space was divided into multiple macrocells, and 3D-DDA traversal algorithm was used to enhance sampling efficiency [19,20].

### 2.2. User Evaluation

To validate the superiority of our system, we implemented three other widely-used rendering approaches. Mesh [9] refers to renderings of meshes extracted from masks, representing a commonly used approach for displaying 3D segmentation outcomes in academic literature. Realistic Rendering (RR) [10] refers to realistic rendering results of original medical data. Ray Casting (RC) [1] is the Ray-Casting results using original data and segmentation results. We engaged a cohort of physicians in a user survey, comprising a total of 15 participants, including 8 radiologists and 7 surgeons. The statistical results of the user survey ratings were presented using a Gantt chart.

### 2.3. Implementation Details

Before training the segmentation model, the input scans were normalized to zero mean and unit variance. The size of each image was 240 × 240 × 150. During training, the patch size was 128 × 128 × 32. The total number of epochs for training was set to 500. The initial value of the learning rate was set to 0.001, which was adaptively adjusted using the ReduceLROnPlateau method. Stochastic gradient descent (SGD) was used as the optimizer. The batch size was set to 4, which included 2 labeled images and 2 unlabeled images. Data enhancement methods included probability-aware weighted random cropping, flipping and rotation. The segmentation network was crafted with PyTorch 1.8 and trained on a GRID V100D-8Q. The segmentation layers of the network are in Table 2.

Our rendering system was implemented using C++ and CUDA, incorporating an embedded Python environment. It operated seamlessly on an RTX 4070 Ti GPU. Realistic DVR capabilities were achieved through C++ and CUDA programming languages.

## 3. Results

### 3.1. Segmentation Metrics and Results

Dice and 95% Hausdorff Distance (HD95) were employed as segmentation evaluation metrics. The segmentation performance of various methods on the testing set is presented in Table 3, with the first row detailing the outcomes for V-Net [23] trained via supervised learning. Additionally, we have implemented several cutting-edge SSL segmentation techniques for comparison, including calibrating label distribution (CLD) [24] and URPC (baseline) [9] in Table 3. The lack of labels in the training set diminished segmentation accuracy across all categories. On 20% labeled experiments, UCPPA gained 72.9% and 80.0% Dice on meningiomas and SNFH respectively, which improved the segmentation results compared to both CLD and URPC. On 40% labeled experiments, the segmentation performance of UCPPA on meningiomas and SNFH is nearly comparable to that of fully supervised V-Net (Meningiomas Dice: 79.5% vs. 80.0%; SNFH Dice: 82.3% vs. 83.0%). Furthermore, compared to URPC, UCPPA achieved superior segmentation performance while maintaining identical computational complexity (FLOPs).

**Figure 3:**
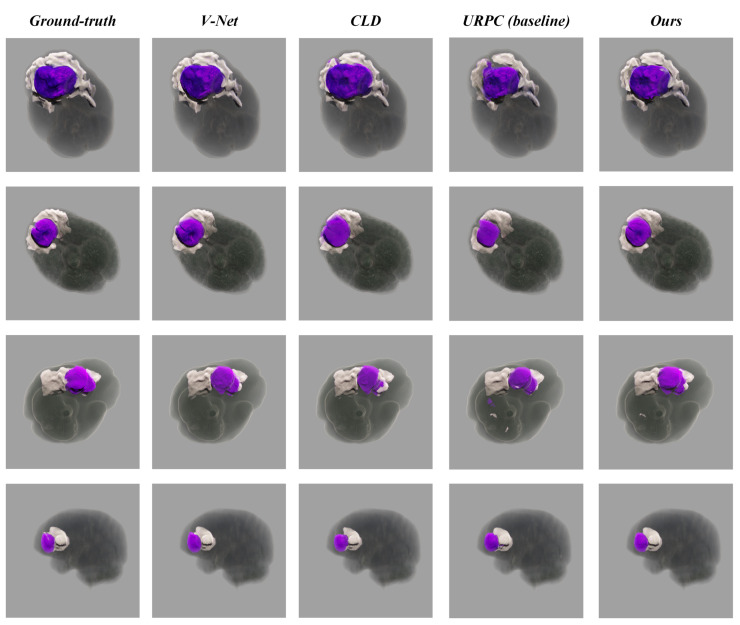
Visualization of different network segmentation results. Compared to the baseline, our network shows superior performance for the segmentation of meningiomas and SNFH (colored in purple and white).

Various segmentation examples from different networks (based on 20% labeled experiments) were visualized using the SegR3D system, as illustrated in Figure 3. UCPPA produced precise segmentation outcomes for both meningioma and SNFH regions. This demonstrates the effectiveness of probability-aware weighted random cropping.

### 3.2. The Role of Importance Transfer Function

**Figure 4:**
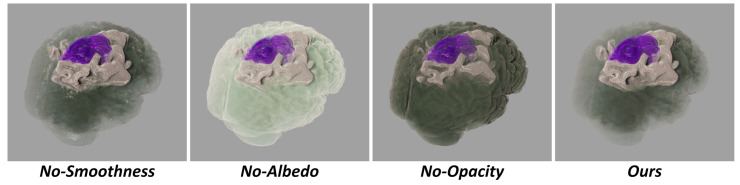
The demonstration focuses on the role of the importance transfer function. No-X signifies that the importance transfer function does not affect X. It can be observed that importance functions play a critical role in rendered appearance.

Lowering the albedo value and smoothness value of unimportant regions helps mitigate the accumulation of highlights, which could otherwise affect observations. Lowering the opacity of unimportant regions aims to prevent them from obstructing important regions. Various visual effects can be created by adjusting the importance transfer function, as illustrated in Figure 4.

### 3.3. User Evaluation of the SegR3D System

**Figure 5:**
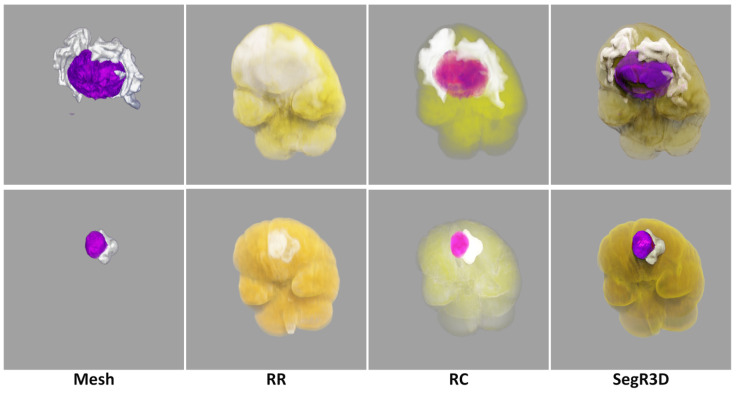
The visualization results generated by different rendering methods. Due to the differing underlying shading paradigms of each technique, there are slight variations in the color of the visual effects.

In user evaluation, we presented four descriptors, and the physicians rated their level of agreement with these descriptions. Ratings ranged from 1 to 5, with 1 indicating “strongly disagree” and 5 indicating “strongly agree.” The first three descriptions are utilized for assessing visualization algorithms, as depicted in Figure 5. The inquiry assesses *Q1* whether our approach is more conducive to perceiving the location and distribution of lesions, *Q2* whether it facilitates better perception of tumor shape, and *Q3* whether it holds advantages over other methods. We exclude methods that require complex manual operations to achieve the desired visualization. The fourth description pertains to assessing *Q4* whether our system’s visualization outcomes have reached a sufficient level of precision for lesion analysis and surgical planning when compared to those obtained from ground truth.

**Figure 6:**
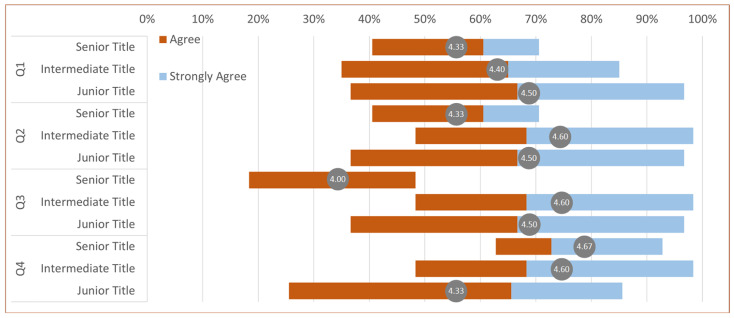
Gantt chart for user experiment rating results.

The statistical results of user experiment ratings are presented in Figure 6 using a Gantt chart. The statistical results (mean/standard deviation) for the four questions are as follows: *Q1* (4.47/0.52), *Q2* (4.53/0.52), *Q3* (4.40/0.51), and *Q4* (4.40/0.51). It can be observed that all participating physicians consider our system as the optimal visualization tool. They can selectively emphasize lesion areas through the adjustment of the importance transfer function, thereby enhancing comprehension of tumor location and morphology. Supplementary Material Video S1 demonstrates the interactive effects of SegR3D.

## 4. Discussion

In this study, we developed a realistic 3D medical visualisation system named SegR3D, which combines a segmentation pipeline and a realistic rendering pipeline. The segmentation pipeline can segment the tumor region and the SNFH region in meningiomas automatically. The realistic rendering pipeline provides an interactive visualisation of the region of interest and allows the user to adjust the target object’s appearance and observation direction. SegR3D helps radiologists to assess the distribution and volume of meningiomas in the brain and facilitates neurosurgeons to make surgical plans.

To reduce the reliance on expert annotations, the segmentation pipeline used a semi-supervised training approach. The proposed UCPPA network achieved results comparable to fully supervised methods V-Net using only 20% of annotated data (Meningiomas: Dice of 72.9% vs. 80.0%, HD95 of 12.8 mm vs. 9.2 mm; SNFH: Dice of 80.0% vs. 83.0%, HD95 of 10.8 mm vs. 9.7 mm). This success can be attributed to the novel probability-aware weighted random cropping we introduced, which enabled UCPPA to focus more effectively on the lesion regions.

Our visualization system employs AI segmentation technology as a pre-classifier to distinguish between diseased and healthy tissue regions. The visualization engine integrates medical image segmentation with photorealistic rendering to effectively highlight regions of interest, such as lesions. The system not only displays the shape of the lesions but also provides a clear representation of their distribution within the surrounding brain tissue. Furthermore, the design of the importance transfer function simplifies user interaction, allowing users to adjust the importance of different tissues to emphasize specific areas. Compared to existing visualization methods, our approach offers significant advantages. Furthermore, through discussions with physicians, we have learned that the majority of user experiment participants indicated their willingness to consider the visualization outcomes of SegR3D in clinical practice. However, they have also expressed a desire for SegR3D to visualize the relationship between tumors, blood vessels, and nerves to mitigate surgical risks. This constitutes a prospective avenue for our future research endeavors.

Although this work used a large publicly available dataset of meningiomas (BraTS2023-MEN), the segmentation model was still trained and evaluated using a single dataset. This may limit the generalizability of SegR3D. Validation in a variety of clinical settings would be beneficial. Additionally, our system was unable to visualise the relationship between tumors, blood vessels and nerves, whose positional relationship is extremely important for surgical planning. Overall, future work will focus on improving the accuracy and versatility of the segmentation model, enhancing the visualization imaging effect, and improving real-time interaction quality, thereby making SegR3D an ideal tool for physicians.

## 5. Conclusions

In this paper, we proposed an interactive visualization system SegR3D for meningiomas that integrates a semi-supervised segmentation pipeline and a realistic rendering pipeline. Considering the relative smallness of meningiomas compared to the brain, we introduced probability-aware weighted random cropping into the segmentation model, substantially enhancing segmentation performance beyond the baseline. To highlight the lesion location in the visualization results, we proposed an importance transfer function to adjust the material parameters by evaluating the importance of different regions. Furthermore, we introduced realistic rendering to enhance the spatial three-dimensionality of the rendered results. Seg 3D has undergone evaluation by multiple clinicians and has been recognized as highly valuable for tumor analysis and surgical planning.

## Figures and Tables

**Table 1:** Patient Demographics of BraTS2023-MEN Dataset.

	Training Set	Validation Set	Testing Set
Patient Count	666	134	200
Age (mean ± SD)	60.1 ± 14.9	60.8 ± 13.8	59.8 ± 13.6
Gender			
Male	194	38	53
Female	464	94	147
n/a ^1^	8	2	0

^1^ n/a represents that the gender of the patient is not applicable.

**Table 2:** The architecture of UCPPA.

Layer Name	Type	Patch Size/Stride	Output Size (C × D × H × W)
**Input**	-	-	1 × 32 × 128 × 128
**Encoder**			
conv1	Conv3D + BN + ReLU	3 × 3 × 3/1	16 × 32 × 128 × 128
maxpool1	MaxPool3d	2 × 2 × 2/2	16 × 16 × 64 × 64
conv2	Conv3D + BN + ReLU	3 × 3 × 3/1	32 × 16 × 64 × 64
maxpool2	MaxPool3d	2 × 2 × 2/2	32 × 8 × 32 × 32
conv3	Conv3D + BN + ReLU	3 × 3 × 3/1	64 × 8 × 32 × 32
maxpool3	MaxPool3d	2 × 2 × 2/2	64 × 4 × 16 × 16
conv4	Conv3D + BN + ReLU	3 × 3 × 3/1	128 × 4 × 16 × 16
maxpool4	MaxPool3d	2 × 2 × 2/2	128 × 2 × 8 × 8
center	Conv3D + BN + ReLU	3 × 3 × 3/1	256 × 2 × 8 × 8
**Decoder**			
up4	Upsampling + Skip + Conv3D	-	128 × 4 × 16 × 16
up3	Upsampling + Skip + Conv3D	-	64 × 8 × 32 × 32
up2	Upsampling + Skip + Conv3D	-	32 × 16 × 64 × 64
up1	Upsampling + Skip + Conv3D	-	16 × 32 × 128 × 128
**Deep Supervision**			
dsv4	Conv3D + Upsampling	1 × 1 × 1/1	3 × 32 × 128 × 128
dsv3	Conv3D + Upsampling	1 × 1 × 1/1	3 × 32 × 128 × 128
dsv2	Conv3D + Upsampling	1 × 1 × 1/1	3 × 32 × 128 × 128
dsv1	Conv3D	1 × 1 × 1/1	3 × 32 × 128 × 128

BN: Batch Normalization

**Table 3:** Comparison between our method with previous methods (Labeled 20% and 40%). All experiments are conducted in an identical setting.

Method	Labeled	Meningiomas		SNFH		FLOPs (G)
		**Dice (%)**	**HD95**	**Dice (%)**	**HD95**	
V-Net [23]	100%	80.0	9.2	83.0	9.7	97.8
CLD [24]	20%	63.3	16.9	77.4	11.9	195.6
URPC [9]	20%	70.4	14.0	79.1	11.1	36.2
UCPPA	20%	72.9 *	12.8 *	80.0 *	10.8 *	36.2
CLD	40%	70.3	13.7	78.9	11.3	195.6
URPC	40%	77.7	11.6	81.0	10.5	36.2
UCPPA	40%	79.5 *	9.8 *	82.3 *	9.9 *	36.2

* indicates statistically significant improvement over all other methods (*p* < 0.05, Friedman test).

## Data Availability

The data presented in this study are available in BraTS 2023 Challenge at https://www.synapse.org/Synapse:syn51156910/wiki/627000 (accessed on 5 October, 2023).

## Supplementary Materials

The following supporting information can be downloaded at: https://www.mdpi.com/article/10.3390/jimaging11070216/s1, Video S1: The interactive effects of SegR3D.
